# The Blurred Lines Between New Psychoactive Substances and Potential Chemical Weapons

**DOI:** 10.3390/toxics13080659

**Published:** 2025-08-01

**Authors:** Loreto N. Valenzuela-Tapia, Cristóbal A. Quintul, Nataly D. Rubio-Concha, Luis Toledo-Ríos, Catalina Salas-Kuscevic, Andrea V. Leisewitz, Pamela Cámpora-Oñate, Javier Campanini-Salinas

**Affiliations:** 1 Escuela de Química y Farmacia, Facultad de Ciencias, Universidad San Sebastián, Puerto Montt 5501842, Chile; lvalenzuelat@correo.uss.cl (L.N.V.-T.); cquintulc@correo.uss.cl (C.A.Q.); nconchar@correo.uss.cl (N.D.R.-C.); 2 Centro de Estudios en Seguridad y Crimen Organizado (CESCRO), Universidad San Sebastián, Santiago 8420524, Chile; ltoledor@outlook.es (L.T.-R.); catalinasalasku@gmail.com (C.S.-K.); 3 Escuela de Derecho, Facultad de Derecho y Ciencias Sociales, Universidad San Sebastián, Santiago 8420524, Chile; 4 Vicerrectoría de Investigación y Doctorados, Universidad San Sebastián, Santiago 8580702, Chile; andrea.leisewitz@uss.cl; 5 Escuela de Química y Farmacia, Facultad de Ciencias, Universidad San Sebastián, Santiago 7510157, Chile; pamela.campora@uss.cl

**Keywords:** chemical weapons, neurotoxic agents, chemical warfare agents, new psychoactive substances, non-lethal chemical weapons, cognitive warfare

## Abstract

The historical use of toxic chemicals to cause intentional harm has evolved from blister agents in World War I to highly lethal organophosphates and emerging families of chemicals, such as Novichok. In turn, medical or recreational substances like fentanyl, lysergamides, and phencyclidine pose a growing risk of hostile use, particularly related to the rapid proliferation of new psychoactive substances (NPSs). A narrative literature review was conducted covering specialized databases (PubMed, ScienceDirect, SciELO, Google Scholar) and sources from international organizations (OPCW, UNODC, ONU), analyzing historical and recent cases of the use of nerve agents in conflicts and the use of NPSs for hostile purposes. The main families of conventional agents (G, V, A series, and Novichok) and NPSs (lysergamides, PCP, fentanyl derivatives) were identified, highlighting their ease of synthesis, high toxicity profiles, and the regulatory gaps that facilitate their illicit production. In this scenario, it is essential to strengthen regulatory frameworks, surveillance systems, and ethical protocols in chemical research, as well as to promote international cooperation to prevent these substances from becoming chemical threats.

## 1. Introduction

Throughout history, chemical weapons have played a pivotal role in some of humanity’s darkest moments, exemplifying the destructive potential of chemistry. The first significant use of chlorine gas occurred during World War I, a choking agent that had caused numerous fatalities by 1915. This was soon followed by the use of mustard gas, an abrasive substance that severely irritated the eyes, skin, and lungs, leading to serious injuries among soldiers [1]. A period of widespread use of multiple chemical compounds in warfare underscored the devastating consequences of chemical weapons, prompting the establishment of the Chemical Weapons Convention in 1993. This treaty aimed to eliminate the production, stockpiling, and use of chemical weapons for military purposes, as well as to dismantle the facilities where they were manufactured [2]. To ensure the correct implementation of this treaty, the Organization for the Prohibition of Chemical Weapons was founded in 1997 [3]. In recognition of its significant efforts to reduce the use, production, and storage of chemical weapons, the organization was awarded the Nobel Peace Prize in 2013 [4]. In addition, this organization is tasked with actively monitoring the peaceful use of chemical sciences.

These sciences play a fundamental role in the technological development of humanity, particularly in the discovery and creation of new drugs for the treatment, diagnosis, and prevention of disease. However, the same substances that are developed for beneficial purposes can also be diverted toward harmful uses. Fentanyl, for example, is an opioid analgesic that is more potent than morphine, which is indicated for the treatment of severe pain. Yet, in the past decade, it has been widely manufactured and distributed as a drug of abuse, leading to hundreds of overdose deaths [5]. Additionally, fentanyl and its derivatives have been used as incapacitating agents, with lethal outcomes. During the Dubrovka Theater hostage crisis in Moscow, the Russian government used a gas based on fentanyl analogs to neutralize the Chechen terrorists who had taken over the venue, with 800 civilians inside. This operation resulted in the death of 33 terrorists and 123 hostages due to the gas [6].

Currently, new synthetic drugs or new psychoactive substances (NPSs), designed to evade regulation, while producing effects similar to or stronger than illegal drugs, pose a significant risk of being used with harmful intent against the population [7]. Many of these substances are highly toxic and relatively easy to produce, increasing the risk of their use for malicious purposes. The United Nations Office on Drugs and Crime (UNODC) has identified over 1200 NPSs that have been reported in the past ten years, reflecting their growing presence in the global market [8]. The rapid development of these substances poses a substantial threat to public health, as they are poorly understood and increase the risk of severe intoxication, addiction, and overdose [9].

Given this situation, it is essential to establish informative, legal, and regulatory measures aimed at preventing the fatal consequences resulting from the widespread use of these substances. This article aims to provide information on substances that may have been designed as pharmaceuticals or drugs of abuse but that pose a significant risk to society if used for military purposes. By analyzing known chemical agents and new psychoactive substances (NPSs) that could potentially be used as chemical weapons, this work seeks to offer bibliographic resources to support the implementation of protective measures. It is crucial to emphasize that the purpose of this study is not to promote the misuse of these substances, but rather to raise awareness of the inherent dangers associated with scientific and technological progress. This underscores the importance of maintaining strict ethical standards in the research and development of new technologies and substances.

## 2. Bibliographic Review

A comprehensive narrative literature search was conducted to evaluate the potential for new synthetic drugs to be used as chemical weapons. This involved investigating existing chemical weapons, their evolution, and their historical impact. The following keywords were used: “History of Chemical Weapons”, “Chemical Weapons”, “Neurotoxic agents”, “Chemical War”, “Warfare agents”, “Cognitive Warfare”, “New psycho-active substances (NPS)”, “Incapacitating agents”, “Fentanyl analogs”, “Synthetic Opioids”, “Lysergamides (LSD)”, “Phencyclidine (PCP)”, “Synthetic Cannabinoids”, “Dark Classics in Chemical”, “Toxicology of synthetic opioids”, “Chemical terrorism”, “Benzodiazepine analogs”, and “Lysergamide derivatives”.

Specialized search engines, such as PubMed, SciELO, ScienceDirect, and Google Scholar, were utilized.

Additionally, international events were reviewed to assess how synthetic drugs can be related to chemical weapons through the assessment of case studies, government reports, and scientific analyses documenting incidents and the potential use of these substances. The keywords used for this purpose included: “Drugs as chemical weapons”, “Psychowar”, “Incapacitating agents”, and “Chemical Warfare and Terrorism”.

Resources from various governmental and non-governmental organization sources and organizations were used, including the Organization for the Prohibition of Chemical Weapons (OPCW), the Chemical Weapons Convention (CWC), the North Atlantic Treaty Organization (NATO), the British Medical Association, the Royal Society, the United Nations (UN) Security Council, and the United Nations Office on Drugs and Crime (UNODC).

## 3. Results

### 3.1. Historical Review of Modern Chemical Weapons

The use of chemical substances in weapons has a long history, dating back thousands of years. From ancient hunting techniques, where arrows or spears were coated with poisons derived from plants, animals, or minerals, to the complex development of chemical agents used in modern-era warfare [10,11].

However, it was during the XIX century, with the advent of the Industrial Revolution and the advancements in modern chemistry, that the ability to manipulate substances and materials more efficiently and precisely reached new heights [11,12]. These advancements, along with the increasing mechanization of warfare, facilitated the development of the first chemical warfare agents, which were deployed during World War I [13].

Thus, choking gases, such as chlorine and phosgene, blister agents like mustard gas and lewisite, and blood agents, such as hydrogen cyanide and arsine, constituted the first generation of chemical weapons. These agents affected more than 1.3 million people, resulting in more than 100,000 deaths in Central and Western Europe [14,15]. This tragic event not only highlighted the magnitude of the use of these chemical agents on the battlefield, but also marked the beginning of a race to develop even more lethal chemical weapons [16].

After the First World War, public opinion strongly condemned the use of chemical weapons, considering them inhumane, due to the impact they had caused. This widespread rejection of such practices led to the signing of the Geneva Protocol in 1925, which banned the use of chemical and biological weapons in international conflicts [14]. However, by this time, chemical warfare had already become entrenched in the military doctrines of various nations. Despite being a significant step forward, the implementation of the Geneva Protocol was weak, as it did not ban the production or stockpiling of such weapons. This allowed for their continued development and accumulation as a preventive measure in anticipation of future conflicts [11,16,17].

Consequently, the development of chemical weapons peaked in the 1930s. The experience with the first generation of chemical weapons showed that, although effective, they were relatively less efficient than initially expected. In response to this limitation, Germany, seeking to optimize the lethality of these agents, began researching organophosphates, pesticides originally designed for agriculture [14,15,17]. These compounds exert their toxic effects by inhibiting acetylcholinesterase (AChE), an essential enzyme for the functioning of the central nervous system (CNS). AChE is responsible for breaking down the neurotransmitter acetylcholine (ACh) in the synapses of the nervous system. By inhibiting this enzyme, organophosphates cause the accumulation of acetylcholine, which leads to the overstimulation of receptors, resulting in paralysis and death. Based on this observation, in 1936, Gerhard Schrader, a German chemist working for IG Farben, synthesized (R/S)-ethyl N,N-dimethylphosphoramidocyanidate, an organophosphate compound that he named tabun (GA). This discovery led to the development of the second generation of chemical weapons and the first nerve agents, known as the G-series, which includes agents such as sarin (GB), soman (GD), and cyclosarin (GF) [13,14].

With the end of World War II and the onset of the Cold War, the United States and the former Soviet Union continued to develop chemical weapons. In 1952, British chemist R. Ghosh, working in the Plant Protection Division of Imperial Chemical Industries (ICI), synthesized the first V-series agent, namely O,O-diethyl S-(2-diethylaminoethyl) phosphorothioate, which is better known as Amiton or VG. Initially marketed as a pesticide, it was later withdrawn due to its high toxicity, and its patent was subsequently sold to the United States [18]. Subsequently, a group of American scientists discovered that by modifying the alkoxy–phosphate bond in the structure of Amiton and replacing it with a phosphorus–methyl bond, the compound’s toxicity increased by a factor of ten [11,18,19]. This led to the development of VE and the beginning of the third generation of chemical weapons, known as the V-series, which includes four main agents: VG, VE, VM, and VX [11,16]. Unlike the G-series, these agents are significantly more lethal, with greater stability and lower volatility, which gives them increased environmental persistence [20].

In response to this, the former Soviet Union, between the 1970s and 1990s, developed an extensive arsenal of new chemical weapons, derived from the G- and V-series, dubbed the A-series. The first compound in the series was N-2 diethylaminomethylacetoamidido-methylphosphonofluoridate (A-230), a sarin derivative in which the O-isopropyl group was replaced by an acetoamide radical [21]. These compounds, such as A-230, A-232, A-242, and A-246, were designed to be much more toxic than their predecessors, with scientific research indicating that their toxicity was 5 to 8 times greater than VX. However, due to their classification under the Foliant Program, a secret Soviet project whose primary objective was to create more powerful chemical weapons that could withstand conventional protective measures, many details about the A-series remain unknown or are difficult to verify [21,22].

Despite the lack of detailed information, it is known that, within this research framework, a new family of chemical weapons emerged in the 1980s, known as Novichok [21]. Although some of these agents are part of the A-series and derived from it, the Novichok family was designed as the fourth generation of chemical weapons. This group includes agents such as A-234 (Novichok-7), A-232 (Novichok-5), A-241, among others, engineered to be more lethal and harder to detect than their predecessors. The strategy behind these agents was to exploit their novelty and limited public knowledge to circumvent the existing chemical weapons control systems of that time [11,22].

**Figure 1:**
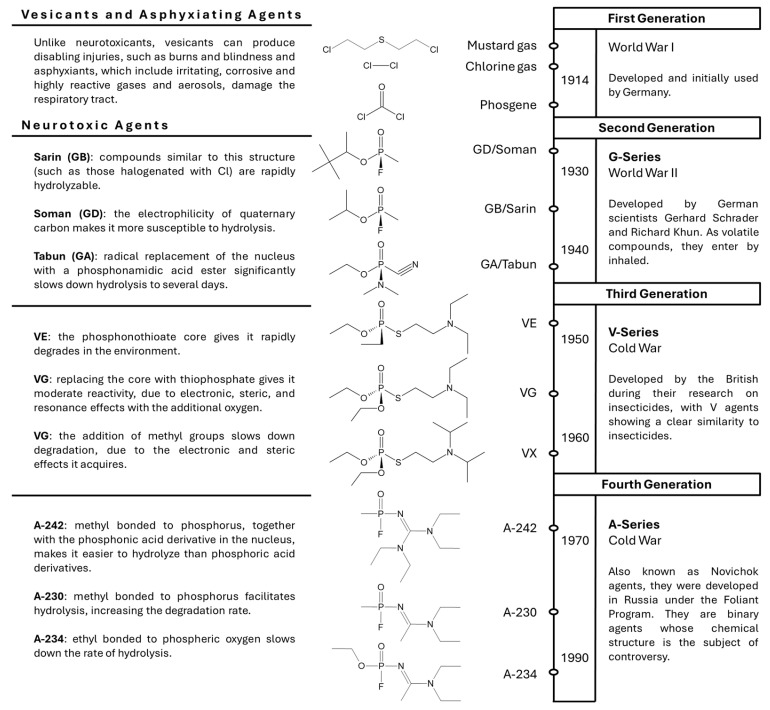
Structural evolution of chemical weapons and their modifications over the years [13,33,34,35].

The evolution of these agents has required the constant modification of their chemical structures, giving them diverse effects as a result. Figure 1 shows the different structural modifications of these agents in the different generations and their influence on stability in the environment, due to their hydrolysis capacity.

Following the historical review of the evolution of modern chemical weapons, it is necessary to quantify the differences in lethality between the various generations of nerve agents. For this purpose, Table 1, below, presents estimates of their relative potency in humans, based on experimental data.

Due to the devastating and often indiscriminate effects of chemical weapons on both the battlefield and in regard to civilian populations over the years, their use led to the signing of the Chemical Weapons Convention (CWC) in 1993, which came into force on 29 April 1997 [16]. The Convention constitutes the first multilateral treaty designed to prohibit the development, production, stockpiling, transfer, and use of chemical weapons, ensuring the destruction of their residues within a specified period of time. The CWC also forms the Organization for the Prohibition of Chemical Weapons (OPCW), whose purpose is to permanently and verifiably eliminate chemical weapons and prevent their future development or proliferation [16,36].

The OPCW defines chemical weapons as “any toxic chemical which can cause death, injury, temporary incapacitation or sensory irritation to humans or animals through its chemical action on life processes”. This definition also includes chemical precursors used to produce toxic chemicals, as well as any munitions, devices, or equipment specifically designed to cause death or harm by releasing such substances [36].

Chemical weapons comprise a wide variety of toxic chemical agents, ranging from industrial chemicals, toxins, and bioregulators to “chemical warfare agents”, which the OPCW groups into five main categories (Table 2).

In addition, the CWC establishes three lists, or “Schedules”, in the Annex on Chemicals (Annex to the Convention), which categorize substances that must be controlled, monitored, or destroyed (See Appendix A, Table A1). This system enables a graduated level of control over chemical substances and their precursors based on their potential use in the production of chemical weapons or other activities prohibited by the Convention. Schedule 1 includes toxic chemicals and precursors that have been developed, produced, stockpiled, or used as chemical weapons. Among them are well-known agents such as sarin, soman, and VX. The criteria for inclusion in this list are based on the structural similarity of these substances to others already listed, as well as their highly lethal or incapacitating properties that make them susceptible to use for warfare purposes (Table A2).

On the other hand, Schedule 2 includes toxic chemicals and precursors, which present a risk due to their toxicity and potential to be used in the production of chemical weapons, either directly or as precursors in chemical reactions that end in the formation of products listed in Schedule 1 or part A of Schedule 2. Examples of these substances are Amiton and BZ and some precursors, such as methyl phosphonyl dichloride (Table A3).

Finally, Schedule 3 groups together toxic chemicals and precursors that have been produced, stockpiled, or used as chemical weapons and that can be produced in large quantities for legitimate commercial purposes. However, the potential for their diversion to prohibited activities justifies their inclusion under monitoring and control measures. Examples include cyanogen chloride and certain precursors, such as phosphoryl oxychloride, sulfur dichloride, among others (Table A4).

However, despite international efforts to eradicate chemical weapons, some countries and terrorist groups continue to use prohibited chemical agents. A notorious case occurred during the conflict in Syria in 2013, wherein the regime of Bashar al-Assad was accused multiple times of using chemical weapons against civilian populations. Specifically, this related to various areas of Ghouta, wherein the use of sarin gas was reported to have caused hundreds of deaths and thousands of injuries. Another incident took place in Salisbury, United Kingdom, in 2018, when a former Russian spy and his daughter were poisoned with the nerve agent, Novichok [16,37].

The use of these advanced agents, highly lethal and difficult to detect, demonstrates that chemical weapons remain a tool of warfare for certain state and non-state actors seeking to evade international regulations [38]. This pattern of ignoring treaties highlights not only the persistence of the use of chemical weapons in warfare, but also how scientific and technological advancements enable the creation of new substances or the modification of existing chemical structures to produce more dangerous compounds.

More recently, new psychoactive substances (NPSs) have emerged as a growing threat, due to their ability to produce significant effects at minimal doses, and the ability to evade international regulations and conventional detection systems through their constant structural modification [38,39].

### 3.2. From Incapacitating Agents to Neuro-Weapons

The use of psychoactive substances in military conflicts is not a recent problem. Various military, security, and police agencies, as well as state authorities responsible for policymaking in several countries, have examined the potential of using or developing chemical substances to alter a person’s perception, cognitive abilities, and behavior in order to temporarily incapacitate adversaries. Countries involved in such research are the United States, the former Soviet Union, South Africa, Albania, China, Iraq, Israel, the United Kingdom, and Yugoslavia [39,40].

The development of modern chemical weapons in the 20th century introduced a new approach to using drugs as weapons. Advances in psychopharmacology and the increase in armed conflicts sparked interest in exploring alternative forms of warfare, beyond traditional methods [41]. This interest, partly driven by emerging international restrictions on the use of lethal chemical weapons, led to the investigation of so-called “incapacitating agents”, also referred to as “non-lethal” chemical weapons [39,41].

The history of these agents dates back to 1948–1950, when the U.S. Army Chemical Corps, along with other military institutions, initiated an active program called the Medical Research Volunteer Program (MRVP). This program was part of the Edgewood Arsenal in Maryland, a facility dedicated to the research and development of chemical weapons from World War I through the Cold War. Between 1948 and 1975, more than 6700 soldiers participated in MRVP trials to assess the lethal and incapacitating potential of more than 250 chemical substances, ranging from nerve agents to psychoactive compounds, such as LSD and cannabinoids [41,42,43,44,45].

In parallel, amidst the potential threat of a nuclear conflict with the Soviet Union, the U.S. Central Intelligence Agency (CIA) directed its research towards the application of new cognitive techniques, considering them as the most powerful resource available for obtaining a strategic advantage [14]. The objective was to identify agents that, when administered, could induce conditions similar to psychotic disorders without causing permanent harm, thereby enabling the temporary incapacitation or manipulation of an adversary’s mind [41].

Within this framework the Project MK-Ultra emerged, authorized by the first CIA director, Allen Dulles, on 13 April 1953. Its goal was to develop techniques and employ methods of mind control and behavioral manipulation through interrogation experiments and the administration of psychotropic drugs, with the aim of using such covert strategies in a potential confrontation [14,46]. However, MK-Ultra never achieved its operational objectives and was terminated in the early 1970s due to multiple factors: the low reproducibility and consistency of the scientific results, methodological deficiencies in its protocols, ethical objections arising from the use of torture and covert practices, and the subsequent order to destroy a large portion of its records [14].

Despite extensive U.S. research on incapacitating agents for potential use as chemical weapons, only 3-quinuclidinyl benzilate (QNB), developed in 1952 by the pharmaceutical company, Hoffman-La Roche, as a promising antispasmodic for the treatment of gastric disorders and ulcers, was ultimately standardized as a weapon [14,41]. The potent anticholinergic activity of QNB aroused the interest of the U.S. Chemical Corps, which decided to study it under the code EA-2277 or BZ. This compound showed an incapacitating concentration per exposure time in air (ICt_50_) via aerosol dispersion of approximately 170 mg·min/m^3^, resulting in various physiological and psychological effects. These effects include a dry mouth, increased heart rate, mydriasis, mild sedation, mental deceleration, confusion, impaired perception, and memory impairment [10].

Although BZ was introduced into the armed forces around 1961 and was produced in quantities totaling 50 tons for use in aerial cluster bombs, its effects proved to be unpredictable. Additionally, it has a lethal concentration per time (LCt_50_) of approximately 200,000 mg·min/m^3^, which is very close to its incapacitating threshold, making it unreliable for battlefield use. Consequently, interest in the agent declined, ultimately leading to the suspension of its research and production [10,41].

Nevertheless, the use of these agents for hostile purposes continues to be at the forefront of research initiatives. Despite chemistry’s significant role in science and technology, enabling the development of innovative drugs for the treatment, diagnosis, and prevention of diseases, these same substances, initially created for beneficial purposes, can be misappropriated for harmful purposes [3,10].

An example of this is scopolamine, a tropane alkaloid derived from plants, such as *Scopolia carniolica* and *Datura stramonium*, which possesses anticholinergic properties. Originally developed for medical use to treat nausea, vomiting, and motion sickness, it is now used for chemical submission, as high doses can induce intense psychoactive effects, such as hallucinations, memory loss, confusion, deep sedation, and even death [41,47].

In this context, the Scientific Advisory Board (SAB) of the OPCW has recently expressed its concern that opiates, specifically fentanyl, used as an anesthetic in surgical procedures, and its analogues, such as carfentanil, used in darts to sedate large animals, could potentially be used for hostile purposes [39,40]. This concern arises from incidents, such as the Dubrovka Theater case in 2002, when Russian Special Forces used a combination of synthetic analogues of fentanyl, resulting in the death of at least 123 hostages and 33 terrorists [39,48,49].

A wide range of observers, including scientific and medical professionals, as well as arms control organizations and international legal experts, are becoming increasingly critical of the potential development and use of these agents, emphasizing that the use of such weapons as incapacitating agents is a matter of concern [38,40].

Although there is no single definition of incapacitating agents, the Royal Society in 2012 defined them as substances whose chemical action affects specific biochemical processes and physiological systems, particularly those related to the regulation of the central nervous system (CNS). These substances induce a state of incapacitation, which may include disorientation, incoherence, hallucinations, sedation, or a loss of consciousness [41,48]. However, although incapacitating agents are sometimes mistakenly designated as non-lethal weapons, in high doses, these substances can cause serious injury or death. For this reason, the North Atlantic Treaty Organization (NATO) refers to them as “neurological weapons” [39,50].

On the other hand, the British Medical Association has concluded that: “The agent by which individuals could be incapacitated without risk of death in a tactical situation does not exist. In such a situation, it is and will remain nearly impossible to deliver the right agent to the right people in the right dose without exposing the wrong people”. This is due, in part, to the variability in how each person responds to the same dose, influenced by factors such as genetics, age, and health status, among others. Therefore, the development and use of these weapons present potentially serious risks to health and well-being [51].

### 3.3. The Use of Neuro-Weapons in Possible Cognitive Warfare

In 2008, the United States National Research Council (NRC) highlighted in its report, “Emerging Cognitive Neuroscience and Related Technologies”, that advances in nanotechnology and gas-phase techniques could enable more effective methods for dispersing chemical agents over large areas [52]. Although it was noted at the time that pharmacological agents are not used as weapons of mass destruction because large-scale deployment is impractical, the report warned that “…*technologies that may be available in the next 20 years would allow for the dispersal of agents by delivery vehicles similar to a pharmacological cluster bomb or a landmine*” [40,48,52]. It also highlighted that, at present, research into microencapsulation has gained importance as a promising technique to enhance the stability, penetration, and controlled release of specific chemical agents, with a particular focus on the delivery of “incapacitating agents” [48].

Since 2017, the term “Cognitive Warfare” has gained relevance in the United States, initially linked to the economic sphere to describe destabilizing actions and influences exerted by companies on consumers, competitors, or legislators [53]. Currently, this concept has evolved and is used by state and non-state actors to refer to actions aimed at “*manipulating the cognitive processes of an adversary or its population, in order to weaken, infiltrate, influence, subjugate or even destroy it*” [54]. This form of warfare is known for its multifaceted nature and varied strategies aimed at influencing human thoughts, emotions, and behaviors. It encompasses a range of tactics from subtle psychological manipulations to overt information operations [53,54].

Since 2021, cognitive warfare has become increasingly significant within international organizations, such as NATO’s Allied Command Transformation (ACT) and NATO’s Science and Technology Organization (STO). According to the ACT, adversaries are enhancing their cognitive warfare capabilities and employing cognitive attacks, offensive actions deliberately directed at the human mind to disrupt the Alliance by affecting perceptions, beliefs, interests, objectives, decisions, and behavior. These strategic analyses highlight the utilization of neuro-weapons as key instruments in cognitive manipulation [38,39].

In the same context, the Biochemical Security Project 2030 and NATO, in their report “Chemicals in Cognitive Warfare: A Peek Inside the Mind-Modifying Arsenal”, outline several concerns about the use of these weapons. These include the risk of “creeping legitimization”; the dangers of their proliferation to both state and non-state actors; their potential use as a lethal “force multiplier”; their use to facilitate torture and other human rights violations; the potential for states to use these agents for law enforcement as a cover for the operation of covert offensive chemical programs; and the risk of their use leading to a new form of chemical warfare [38,40]. Even though the development of these agents is restricted by a comprehensive legal framework, the perceived ambiguity within the CWC could, according to certain interpretations, allow room for their development. This presents a considerable risk given the advancements in science and neuroscience, which are opening up new pathways for chemical weaponry without adequate control [38].

### 3.4. The NPS Phenomenon

New psychoactive substances (NPSs) are substances of abuse not controlled by the 1961 and 1971 Conventions on Narcotic Drugs and Psychotropic Substances, which may threaten public health. Since their emergence, they have been known as “designer drugs”, “legal highs”, and/or “herbal highs”. Similar to the creation of new substances for warfare purposes, the introduction of NPSs into the market is influenced by slight modifications to their chemical structure [55]. The spread and production of these substances is a multifactorial phenomenon that involves the search for alternatives to traditional drugs, the economic gain from synthesizing more potent drugs at lower doses, and the evasion of legal controls by commercializing substances not classified under the law [56].

This emergence of NPSs at high speed and in large quantities poses a considerable risk to public health and a legislative challenge for regulatory bodies, due to the limited history of these substances and the scarce toxicological information available, with these substances being associated with side effects such as convulsions, aggression, psychosis, and dependence, including severe intoxications that require hospitalization [55]. As of 2023, a total of 1250 NPSs have been reported worldwide through the Early Warning System (EWS) on NPSs, making it a global phenomenon affecting 147 countries and territories across all regions [55,57].

New psychoactive substances (NPSs) comprise a diverse group of drugs that possess functional similarities to traditional drugs. These substances pose considerable risk to public health due to their availability on the illicit market. Furthermore, the diverse effects that NPSs can have on humans are comparable to the physiological and cognitive-altering capabilities of chemical weapons. For this very reason, several of these drugs were initially studied for military purposes or have even been previously used with harmful intent against populations. In fact, certain substances developed for either of the two purposes, recreational drug use or chemical warfare, share a fundamental characteristic, namely their potential to incapacitate the human body due to the magnitude and rapid onset of their chemical action, a process influenced by both the dose and potency of the substance involved.

Considering the development of NPSs that are even more potent than the substances from which they are derived, concerns arise not only regarding their recreational use, but also their potential application for harmful purposes against the civilian population. Along with this background, the illicit market has facilitated the acquisition and synthesis of illicit substances, especially through communication channels such as the “Dark Web”. The recently closed website, AlphaBay, was a digital black market that had more than 250,000 advertisements related to the sale of illegal drugs and toxic chemical substances, including powerful synthetic opioids, such as U-47700. The site was uncovered after being linked to a fatal fentanyl overdose, as the substance had been obtained through this platform [58].

Currently, NPSs are not included in the control Schedules of the Chemical Weapons Convention, as they are generally regarded as substances intended solely for recreational use. However, bibliographic evidence related to psychoactive substances highlights the potential of these NPSs to cross the thin line between an illicit drug and a chemical weapon. Based on this premise, we present background information on certain families of NPSs that have had harmful global impacts, along with a detailed description of their potential for use for malicious purposes.

#### 3.4.1. Lysergamides

**Figure 2:**
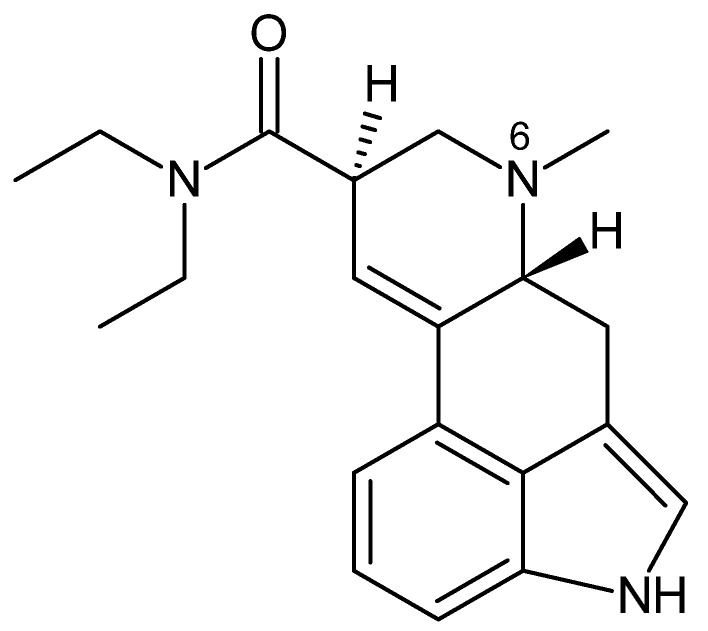
Chemical structure of LSD. Position 6 is marked because of its relevance to the structure-activity relationship of the molecule.

Lysergamides are alkaloids, derived from the chemical compound ergoline, molecules from the ergot fungus [59], of which the best known is LSD-25, or simply LSD (Figure 2). In the 1930s, the Swiss pharmaceutical company, Sandoz, began investigating compounds from a fungus growing on rye and other cereals as possible circulatory stimulants [10]. During these studies, in 1938, chemist Albert Hofmann, working for Sandoz, synthesized several compounds based on the chemical structure of ergot alkaloids, discovering lysergic acid diethylamide (LSD) [12].

In 1943, Hofmann discovered that LSD possessed extremely potent effects, being 4000 times more intense than mescaline, the most potent psychedelic known at the time [10]. This finding led to the evaluation of LSD for treating psychiatric disorders, such as schizophrenia and neurosis [60,61]. Consequently, during the 1940s, LSD was used in numerous clinical studies to explore its ability to induce altered states of consciousness that could facilitate the comprehension of mental and emotional processes [12].

LSD was initially marketed as Delysid^®^ in 1947, with the indication of relieving anxiety states and obsessive neuroses. However, it was eventually withdrawn from the market as its use expanded as a recreational drug during the hippie movement, and it was declared illegal in the United States in 1966 [62]. Recently, “research chemicals” have appeared on the market in the form of various LSD derivatives, many of which had never been described in the scientific literature at the time, as they were previously unknown substances [59]. Among the psychedelic analogues of LSD is the potent LSZ derivative. In 2015, 1P-LSD was introduced as an alternative to LSZ following its prohibition, exhibiting slightly higher potency than LSD [63].

Users report that these two substances produce effects similar to the original drug, from hallucinations, empathy, and euphoria to altered perception [63]. These two drugs are not considered to have addictive potential and have very low toxicity, making them popular because of their apparent safety profile. However, individuals who report having experienced overdoses have described excessive nausea. Due to these characteristics, they remain popular among consumers, who use them for spiritual, introspective, or recreational purposes [63].

As of 2024, 16 substances in this category have been reported, accounting for 9.5% of hallucinogenic-type substances reported by the UNODC [64]. The structural complexity of LSD limits the extent to which modifications can be made without eliminating or reducing its activity. Significant structural changes are found in the N-allyl derivatives at position 6, acquiring approximately twice the potency of LSD [65].

Following its synthesis, between 1959 and 1965, LSD attracted considerable interest as a potential non-lethal chemical weapon due to its psychedelic effects, which could be triggered even at low doses. During this period, the United States Army Chemical Corps conducted tests to evaluate its effectiveness as an incapacitating agent, under the code EA 1729 [13,66]. Because of its physicochemical characteristics, LSD can be efficiently disseminated as a powder, liquid, or inhalable aerosol [13,66].

Studies conducted by the U.S. Army Chemical Corps revealed that the ICt_50_ of LSD in aerosol form was 30 to 55 mg·min/m^3^ [10,66,67]. However, no data have been published on its LCt_50_.

While in humans there are no direct experimental data on the LD_50_ of LSD, historical and clinical evidence suggests that the lethal dose lies in the range of several tens of milligrams, significantly higher than the incapacitating or recreational dose. An analysis of near-fatal overdose cases estimated the human LD_50_ to be approximately 0.2 mg/kg, which corresponds to a total of ~14 mg for an average 70 kg adult [68]. Other authors have suggested higher estimates based on rodent data and observed blood concentrations, such as 100 to 400 mg administered orally (p.o.), as a theoretical human lethal dose [13,67,69].

The first symptoms of exposure are usually somatic and include mydriasis, dizziness, weakness, drowsiness, nausea, and paresthesias, which manifest within a few minutes after dosing, either orally or via inhalation. Over time, exposure causes profound changes in auditory and visual perception, a loss of a sense of time and space, as well as alterations in mood, thoughts, judgment, and memory [39,66]. Despite the initial results, practical difficulties, such as the unpredictability of its effects, which vary significantly between individuals, the influence of user motivation, and challenges related to the handling and production of LSD, limited its development as a chemical weapon [39,66]. Nevertheless, the interest in using psychedelic substances differently, as tools of war, persisted.

Under the direction of leading psychiatrists, the U.S. Army and the Central Intelligence Agency (CIA) conducted numerous experimental programs involving various chemical agents [70]. One of the best-known programs was MK-Ultra, which was developed during the 1950s. Its main objective was to employ methods of mind control, behavioral manipulation, and techniques for use in a potential cognitive warfare scenario [70,71,72]. In July 1953, when LSD was not yet illegal, a sub-project of the MK-Ultra program was approved for the purpose of investigating the biochemical, neurophysiological, sociological, clinical and psychiatric aspects of LSD. The project involved administering LSD and other agents, such as mescaline, scopolamine, PCP, heroin, and amphetamines, to various groups of people, including prisoners, clients of sex workers, and even CIA agents themselves [71]. However, due to the adverse effects, deaths, and the serious ethical implications, the project was abandoned and the related documents were destroyed [73,74,75].

In the 1960s, investigations on the effects of LSD on perception, consciousness, and reality led to its widespread adoption as a recreational drug within the psychedelic movement in the United States and Europe [60]. This period also witnessed the so-called “countercultural movement”, which contributed to the popularization of the term “psychedelic” as a general category encompassing LSD, psilocybin, and numerous other related drugs. Consequently, the unregulated use of these substances generated several health and safety issues. Public concerns about the associated risks of LSD and other psychedelics became significant [12,60]. In response to the growing abuse, as well as the illicit manufacture and distribution of LSD, the United States classified LSD as a Schedule I substance under its national drug control legislation in 1965, imposing strict restrictions on its production, distribution, and possession, and limiting its use to research contexts [76]. Subsequently, the 1971 Convention on Psychotropic Substances also included LSD in Schedule I. Other jurisdictions adopted similar measures, reflecting global concern about the risks associated with LSD [39,61,76].

#### 3.4.2. Phencyclidines

Also known as “angel dust”, phencyclidine is a hallucinogen capable of inducing euphoria, feelings of omnipotence, superhuman strength, and heightened social and sexual performance [77]. Phencyclidine was one of the first man-made drugs of abuse to be named “America’s most dangerous” in 1970 [78].

In 1926, the pharmaceutical company, Parke-Davis, succeeded in synthesizing phencyclidine, chemically known as 1-(1-phenylcyclohexyl)piperidine (PCP) [79]. After extensive studies, the company began marketing PCP in 1950 under the name Sernyl^®^. This compound was initially designed as an anesthetic and analgesic, noted for its ability to produce rapid and potent anesthesia without causing respiratory depression, unlike other anesthetics available at the time [67,79].

**Figure 3:**
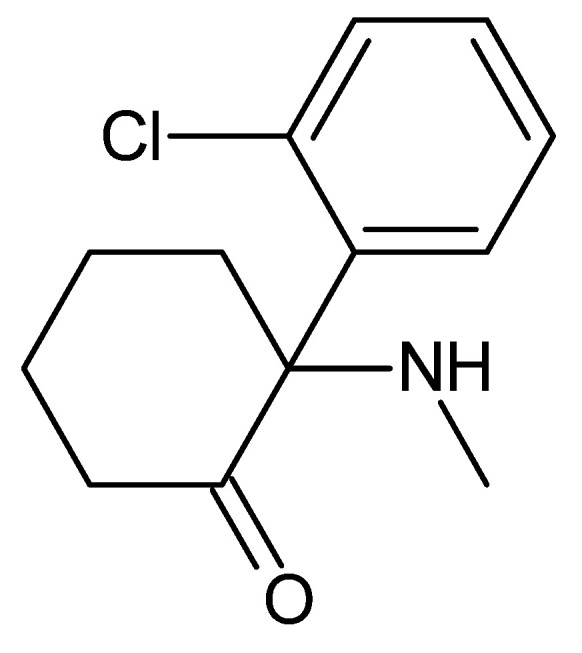
Chemical structure of ketamine.

Although early human trials showed PCP to be safe and effective, clinical testing and hospital use revealed that it produced troubling side effects. This included dissociation, in which patients experienced a disconnection from both their environment and their own body, generating hallucinations, psychosis, and delirium, often resulting in behavioral issues and distressing experiences for patients [78,79,80]. In addition to causing significant cognitive deficits, PCP can also cause tachycardia, increased blood pressure, nystagmus (involuntary eye movement), acute rhabdomyolysis (the death of skeletal muscle fibers and their release into the bloodstream), myoglobinuria (myoglobin in the urine due to rhabdomyolysis), seizures, neurotoxicity, and death [67,78]. An attempt to explore other applications of this substance was made by investigating its possible use as an animal anesthetic. However, PCP was withdrawn from clinical trials because it generated psychosis, agitation, and dysphoria [81]. Subsequently, a structurally related anesthetic, ketamine (Figure 3), was developed and was the only such drug approved for veterinary use, and is often used as a drug of abuse [77].

By the 1970s, PCP had become a widely recognized drug of abuse. Its availability in various forms, tablets, liquids, and solids, and its easy preparation by clandestine chemists led to its indiscriminate use [80]. Over 60 analogs of PCP, more toxic than PCP itself, were created, escaping legal controls. These analogs were widely available in the United States, until they were declared illegal in 1978 [77]. Currently, phencyclidines are relatively inexpensive to produce, and their cost has remained consistently low since the 1980s, contributing to their continued popularity on illicit markets [78]. The simplicity of synthesis by suppliers has resulted in approximately 14 new variants being available as “research chemicals”, which are easily accessible via the internet [78]. These substances are found in a variety of preparations, including tablets, powders, and even mixed within marijuana cigarettes, referred to as “bad tobacco” [77].

As of 2024, there are 34 reported NPSs in this family, representing 89.5% of dissociative-type substances reported by the UNODC [64]. Structural modifications follow certain trends, with the cyclohexyl ring being an essential element in terms of their activity and the tolerance to changes in structure is found in substitutions on the aryl ring and replacement of the heterocyclic ring [78].

In the 1960s, the U.S. Chemical Corps considered phencyclidine as a possible incapacitating agent under the code EA 2148 and codename SN [10,80].

Tests have shown that aerosolized PCP can induce anesthetic effects at concentrations between 25 and 50 mg·min/m^3^, while the estimated ICt_50_ is approximately 1000 mg·min/m^3^ to achieve an effect capable of eliminating activity or disabling combat capability. However, no experimentally defined ICt_50_ value in humans or animals beyond these historical military figures has been reported in the open literature [10]. On the other hand, even if specific information on its LCt_50_ is scarce, available studies provide at least a benchmark in laboratory animals. For example, Sirchie’s NARK2009 safety report indicates that when exposing male rats to PCP in the form of dust or mist for 4 h at a concentration of 0.96 mg/L (equivalent to 960 mg/m^3^), 50% of the animals die after a 14-day follow-up [82]. The LD_50_ values for PCP vary widely by species and the route of administration in rodents, with typical ranges including 16 mg/kg intravenously (i.v.) to 77 mg/kg orally in mice, and 135 mg/kg orally in rats [83].

PCP was considered to be a means of riot control through the use of dart guns loaded with this substance. Originally, these guns were designed to immobilize animals using tranquilizers, causing sedation [78,79,80]. The idea was to adapt this equipment for maintaining public order and safety. However, this approach raised serious concerns, due to the risks associated with its application and the potential for abuse of the substance. The ability of PCP to induce severe and disorienting effects in humans, which, in certain cases, varied depending on the individual, sparked a debate about its safety and efficacy in uncontrolled situations, which contributed to the decision not to pursue its use in regard to these applications [78,79].

As already mentioned, PCP became a widely known drug of abuse in the 1970s. Its availability in various forms (tablets, liquids, and solids) and its easy preparation by clandestine chemists led to its indiscriminate use [80]. Sensationalist media coverage and reports of violent and disruptive acts associated with PCP led to it being declared the “most dangerous drug in America” at the time [78].

In recent years, the media has reported several cases of PCP-induced violence. Reports of abuse of this substance have highlighted extremely violent acts, including cannibalism, murder, kidnapping, and poisoning. For example, in 2003, rapper Big Lurch (Antron Singleton) was sentenced to life in prison after being found guilty of murdering his girlfriend and committing cannibalism under the influence of PCP [78,84]. In 2013, former NFL player, Aaron Hernandez, and two accomplices were convicted of first-degree murder. During the trial, Hernandez claimed that he witnessed his accomplices commit the murder during a violent outburst caused by PCP use [85]. On the other hand, on the night of 8 August 1996, the *Titanic* film crew, led by James Cameron, experienced an unusual incident in Halifax, Nova Scotia. During a celebratory dinner, more than 80 crew members were intentionally drugged with phencyclidine (PCP), which had been added to the cocktail they were drinking. The effects of the drug were so severe that many of those affected had to be taken to Dartmouth General Hospital, where a toxicological analysis confirmed the presence of PCP in their bodies. Following this incident, the Halifax police conducted an exhaustive investigation for more than two years, carrying out searches and interviews, but due to a lack of conclusive evidence, the case was closed in 1999 [78,86].

In addition to its use as a drug of abuse, PCP has also been utilized to adulterate other substances, due to its low cost and ease of manufacture. It is common for PCP to be added to other drugs, especially marijuana, to increase their psychotropic effects and, in turn, their potential for abuse. This practice not only increases the risk of severe adverse effects in users, but also makes it more difficult to identify the actual substance consumed, thereby increasing the dangers associated with its use [78].

In response to the growing abuse and adverse effects associated with phencyclidine (PCP), the United States listed it in Schedule I under the Controlled Substances Act in 1978, pursuant to the Drug Control Act. This classification imposed severe restrictions on its production, distribution, and possession, limiting its use exclusively to research contexts [87]. Similarly, the 1971 United Nations Convention on Psychotropic Substances also included phencyclidine in Schedule II of controlled substances, reflecting global concern about the risks associated with its abuse and dissociative effects [76]. Other jurisdictions have adopted comparable measures to control phencyclidine, reflecting an international consensus on the need to restrict its availability and use to prevent public health problems related to its abuse [39,61].

#### 3.4.3. Fentanyl Derivatives

**Figure 4:**
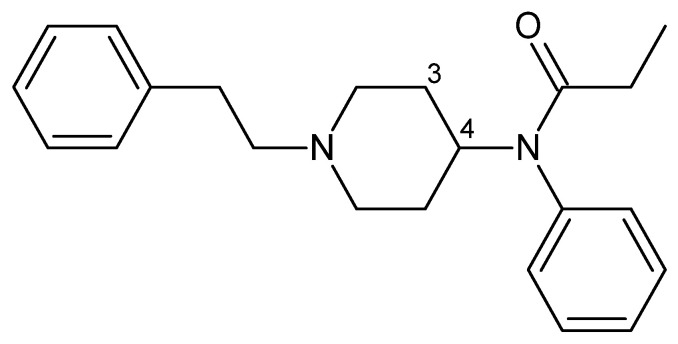
Chemical structure of fentanyl. Position 3 and 4 is marked because of its relevance to the structure-activity relationship of the molecule.

Fentanyl (Figure 4) is an opioid analgesic and anesthetic that is 30 to 50 times more potent than heroin. It was initially synthesized in 1960 and approved for the treatment of moderate-to-severe pain [88]; many analogues were developed for veterinary and medicinal use following its creation [89].

The isolation of morphine and its potential as an analgesic were quickly recognized, leading to its use in a variety of medical applications. The need to control pain on the battlefield promoted the widespread adoption of morphine, resulting in a high incidence of addiction among veterans and a significant public health problem. As medicine advanced, the need arose to develop even more potent and specific opioids for pain management [90,91]. In this context, fentanyl emerged as a crucial advancement. First synthesized in 1960 by Belgian chemist Paul Janssen, fentanyl was designed to offer superior pain relief, with significantly greater potency than morphine, being approximately 100 times more potent [91,92]. However, its high potency made it a health concern because it was more addictive than other known opioids [92].

Frequent use of this substance mainly affects the central nervous system, as well as gastrointestinal, cardiovascular, and pulmonary systems, with respiratory depression being the most dangerous reaction, due to its lethality. Its use is a growing threat to public health in countries such as the United States [88].

The increase in physicians prescribing opioid drugs contributed to a significant escalation of overdose deaths in the United States, exceeding 60,000 deaths in 2016 from the deliberate prescription of these drugs during those years [6]. In 2018, nearly 170 million prescriptions for opioids were issued, which represented a pre-subjectable rate of 51.4 prescriptions per 100 people [93], according to data from the U.S. Centers for Disease Control and Prevention (CDC). After that peak, prescriptions began to decline progressively as a result of new control policies and medical awareness. This reduction, although necessary, was accompanied by a worrying shift towards the use of illicit opioids, such as heroin and, later, fentanyl and its analogues. According to the CDC, synthetic opioids, primarily fentanyl and its derivatives, are the leading cause of overdose deaths, representing an overall increase of 66.1% from 2017 to 2021 [6,94,95]. By 2021, more than 106,699 people had lost their lives due to excessive opioid use [96]. Fentanyl and its illicit derivatives are easy and inexpensive to manufacture and are mainly imported from clandestine laboratories in China and Mexico [6]. According to reports from the DEA and the U.S. Congress, they were often imported into U.S. markets from the Orient via international postal services and dark web channels [6,95]. However, after 2020, both China and the U.S. have implemented anti-drug trafficking policies aimed at decreasing fentanyl imports into U.S. territory [6,97,98,99].

This substance and its analogues are much more commonly found as adulterants or substitutes for opiates of abuse in Europe and North America [6]. They are often mixed with fillers (lactose, mannitol, and sugars) and are commonly used as an adulterant to ‘cut’ street heroin to increase the potency of the product and improve the economic yield. These combinations are extremely dangerous, as fentanyl is so potent that even tiny amounts of the substance are considered lethal [100]. In 2014, the first pills containing fentanyl, analogues, and other illicit synthetic opioids, such as U-47700, were detected [89].

The reported NPSs in this family number 86 substances as of 2024, representing 63.7% of opioid-type substances reported by the UNODC [64]. Modifications in the chemical structure mainly influence the piperidine ring segment. In fact, the expansion or contraction of this segment considerably decreases the antinociceptive potency, and the inclusion of groups in position 3, such as carbomethoxy, or groups longer than methyl, reduce the analgesic potency and duration of action, respectively [100,101]. Conversely, the introduction of methyl into piperidine increases the potency, but the most significant change is found with the inclusion of the methyl ester fraction at position 4 of the ring, increasing the potency up to 100-fold and resulting in the creation of one of the most potent opioids on the market, carfentanil [100].

Although fentanyl and its derivatives are not classified as weapons of mass destruction (WMD) in the conventional sense, nor strictly regulated as such in the Chemical Weapons Convention (CWC), their high potency and ability to cause devastating effects at very low doses have led to significant concern about their potential uses for hostile purposes, representing a significant risk in situations of terrorism or covert attacks due to their ability to cause rapid incapacitation and death at low doses, even presenting lethal doses comparable to those of the nerve agent, VX [102,103].

In October 2002, a group of 53 armed terrorists took 979 hostages at the Dubrovka Theater in Moscow [6,92]. Russian Special Forces used a combination of synthesized fentanyl analogues (kolokol-1) that they had developed in order to take on the terrorists, with the aim of sedating them and rescuing the hostages. However, although it was effective against the terrorists, the intervention with these agents resulted in the accidental death of at least 123 hostages and 33 terrorists. In 2005, Russian Special Forces again used an unidentified chemical spray against terrorists holding hostages in a local business, with equally devastating results [39,48,49].

One of the main Russian arguments for these accidental deaths is that they were used without prior operational experience, in an unusual situation and under inappropriate conditions. Initially, the Russian authorities did not want to reveal the compounds used and attributed the deaths of the hostages to the conditions in which they were held during three days of captivity. However, media and political pressure, both nationally and internationally, eventually led Russian Health Minister Yuri Shevchenko to confirm, four days after the event, that the spray used contained fentanyl derivatives. However, the Russian authorities did not specify the exact composition and argued that the compounds were not lethal and, therefore, that they did not consider them to be prohibited by the CWC [6]. It was not until 2012 that the UK Defense Laboratory confirmed, after analyzing samples from that situation, the presence of carfentanil and remifentanil, two potent derivatives of fentanyl [92].

Although no specific ICt_50_ and LCt_50_ values have been established for fentanyl, Manral et al. [104] evaluated its inhalation toxicity in mice using an aerosol. In this study, it was determined that the concentration that caused 50% mortality in exposed mice (LC_50_) was 8.8 µg/m^3^, while the concentration that reduced the respiratory rate by 50% (RD_50_) was estimated at 6.4 µg/m^3^. Since the study shows that fentanyl does not produce sensory or pulmonary irritation, and the RD_50_ and LC_50_ values are very close (indicating a low margin of safety), the authors concluded that this type of substance should not be used as an incapacitating agent [104]. However, based on the toxicological parameters described, it is evident that this drug could be used as a lethal chemical weapon, due to its ability to induce respiratory depression.

Finally, the LD_50_ in animals is well-characterized. In rats, it is around 3.1 mg/kg when administered intravenously; in monkeys, it is 0.03 mg/kg; and in mice, it is 27.8 mg/kg (oral administration) and 6.9 mg/kg (intravenous administration) [105]. There is no experimentally established LD_50_ value in humans for ethical reasons. However, based on clinical reports, forensic studies, and reports by government agencies, it is estimated that a lethal intravenous dose in non-tolerant individuals ranges from 0.007 to 0.015 mg/kg, meaning approximately 0.5–1 mg for a 70 kg adult, and that total doses of ≈2 mg are considered potentially lethal for most of the population [104,105,106,107].

In the case of carfentanil, no standardized ICt_50_ or LCt_50_ values have been defined in humans. However, animal studies show that concentration–time (Ct) values in the order of tens of mg·min/m^3^ trigger serious effects. For example, Gao et al. [108] exposed rats to aerosolized carfentanil at doses of 4 mg/m^3^ for 10 min (Ct = 40 mg·min/m^3^) and observed 100% mortality of the animals, with respiratory arrest approximately 7.7 ± 0.6 min after inhalation began, while at 2 mg/m^3^ (Ct = 20 mg·min/m^3^) no deaths were recorded, concluding that in this model the LCt_50_ would be between 20 and 40 mg·min/m^3^ [108,109].

As for the LD_50_, intravenous administration of carfentanil in mice has been estimated at approximately 18.75 mg/kg, while pharmacokinetic extrapolations and forensic reports suggest a lethal dose for humans would be around 0.3 µg/kg, intravenously [106,110,111,112,113,114,115].

Carfentanil is 20 to 100 times more potent than fentanyl and about 10,000 times more potent than morphine, so its use is restricted to veterinary medicine for large animals [6,116]. Even so, since 2016, it has been increasingly detected as an adulterant in other drugs of abuse in the United States and Canada, due to its synthetic availability and ease of importation [117]. In 2018, the Drug Enforcement Administration (DEA) published a report warning about the detection of fentanyl and its analogues in cocaine seizures, cautioning about the serious risk posed by its inadvertent consumption [92].

Opioids are regulated by the Single Convention on Narcotic Drugs (1961), but depending on their abuse and medical use, they are classified in different schedules. Specifically, fentanyl is listed in Schedule IV, which includes substances with a high potential for abuse and risk to public health, but which also have legitimate medical applications. This means that their production, distribution, and use must be strictly controlled and regulated [76].

#### 3.4.4. Benzodiazepines

Benzodiazepines (BZDs) are prescription drugs that are used to treat stress and anxiety in patients with anxiety disorders [118]. According to the World Health Organization, BZDs are essential drugs for the treatment of various clinical disorders, including epilepsy, phobias, aggression, insomnia, and others [119].

The first BZD entered the US market in 1960, namely Chlordiazepoxide, and was the first of its class to be approved for clinical use. Currently, BZDs have gained popularity due to their broad safety profile, particularly the reduced incidence of respiratory depression compared to older drugs, such as barbiturates [120]. However, for decades there has been concern about this group of drugs, due to their addictive, dependency-producing, and even abusive nature [118]. In 2010, BZDs were associated with 408,021 emergency room visits, accounting for one-third of all visits related to drug abuse and misuse [120].

The phenomenon of BZD abuse is reaching alarming levels, especially when combined with other CNS depressants, such as frequent mixing with opioids (54.2%) and alcohol (24.7%). The combination of other drugs with BZDs is used to enhance the recreational effects and reduce undesirable side effects, such as insomnia and withdrawal caused by stimulants [120]. However, BZDs can cause mild respiratory depression, which, when combined with opioids or alcohol, can aggravate the user’s condition by enhancing respiratory depression [76,120]. The involvement of BZDs in opioid-related deaths increased dramatically from 18% in 2004 to 31% in 2011, and alcohol has been involved in one in four emergency room visits because of BZD abuse and in one in five BZD-related deaths. Moreover, the dose of BZD consumed tends to be much higher when they are combined with other substances than when they are used alone [120].

In addition to these problems, it is now possible to find “designer BZDs” on the market. These substances are manufactured by modifying the basic structure of BZDs and have not been tested as medicines, posing a potential risk to consumers’ health [119]. This situation is further aggravated when considering the existence of substances marketed as a specific BZD, which are counterfeits of other BZDs that differ in terms of their pharmacological activity from the original substance. A case of this occurred in the United States in January 2017, when the director of an online pharmacy was sentenced to eight years in prison for distributing flubromazolam instead of alprazolam. Similar cases of drug substitution have been reported in Finland, Singapore, and Malaysia [119].

The reported NPSs of this family are 35 substances as of 2024, representing 79.5% of sedative/hypnotic-type substances reported by the UNODC [64]. Within the modifications of the core structure, it has been found that the presence of an electronegative group at position 7 determines the activity of the BZD; in the 1,5-BZDs, chloride determines the anticonvulsant and soporific activity; while nitro determines the hypnotic activity [119].

Benzodiazepines are known for their therapeutic use in the treatment of disorders such as anxiety and insomnia, but they have also been used in criminal contexts because of their potent depressant effects on the central nervous system, due to their ability to induce sedation and amnesia in people [119]. However, beyond their misuse, benzodiazepines have aroused the interest of scientists in regard to their application as tools within “non-lethal” chemical weapons. Although not yet standardized or formally used as such, benzodiazepines have been studied for their properties that could be exploited in this context [39].

Between 2005 and 2007, Czech scientists conducted studies on the potential of various pharmacological substances, including benzodiazepines, to be used as “non-lethal pharmacological weapons”. Among their findings, they demonstrated the efficiency of benzodiazepine combinations, such as midazolam, in situations requiring rapid and effective immobilization without causing “permanent” harm in high-risk settings [40].

In a paper presented by Fusek, Hess, and Schreiberova at the 3rd Ettlingen European Symposium on Non-Lethal Weapons in May 2005, the authors detailed how they administered various pharmacological combinations to rhesus monkeys with the aim of finding the dose and combination that would cause “fully reversible immobilization”. However, no exact dose was found for humans without generating permanent damage [40].

**Figure 5:**
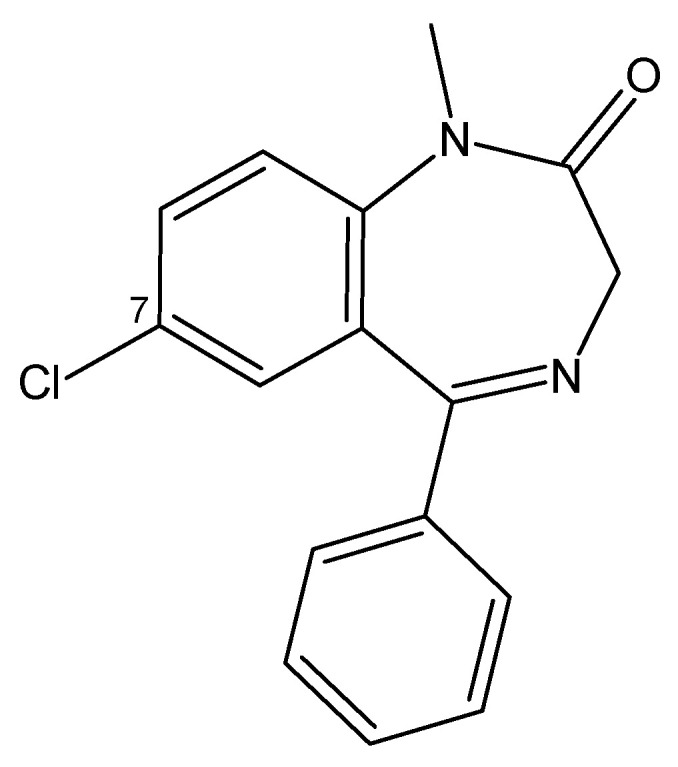
Chemical structure of diazepam. Position 5 is marked because of its relevance to the structure-activity relationship of the molecule.

On the other hand, in the criminal field, flunitrazepam, commercially known as Rohypnol and manufactured by Roche in Basel, Switzerland, is a benzodiazepine that stands out for being ten times more potent than diazepam (Figure 5). Because of its dangerous effects, such as disinhibition, anterograde amnesia, and the elimination of volition, it has been nicknamed “the forgetting pill” or “the rape pill” [119,121]. One case of harmful use occurred in Brussels in 2003, when flunitrazepam was used to intoxicate drunken travelers so that their valuables could be stolen. The case revealed that at least 16 people had positive blood tests for flunitrazepam, at concentrations between 21 and 75 mg/L [121].

Over the years, benzodiazepines have been linked to cases of poisoning and crimes, being the preferred substances of serial killers; one of the most iconic events is known as the “oral hygiene” case, in which, between 1983 and 1987, nurses at Lainz Hospital in Austria used BZD or insulin to murder 43 patients, mainly older adults, by administering them in mouthwashes, killing the victims within a maximum of one hour [122].

Benzodiazepines are increasingly being used in combination with opioids, which has led to an alarming increase in mortality rates. According to the United Nations Office on Drugs and Crime (UNODC), in the United States between 1999 and 2013, deaths related to benzodiazepine use in the United States increased by 514%, and deaths associated with mixing opioids and benzodiazepines reached numbers exceeding 175,000 people [123]. This dangerously increasing pattern of use also extends to the black market, where designer benzodiazepines have gained popularity. Between 2019 and 2020, almost half of the post-mortem analyses in which benzodiazepines were detected involved designer substances, such as flualprazolam, flubromazolam, and etizolam, which are particularly difficult to trace [124]. In addition to this, a scientific review compiled 49 reports in which the cause of intoxication, conduction disturbance, and death were related to BZD, mentioning 3-hydroxyphenazepam, adinazolam, clonazolam, etizolam, deschlorotizolam, diclazepam, flualprazolam, flubromazepam, flubromazolam, meclonazepam, phenazepam, and pyrazolam [119,124].

Globally, there has been a significant increase in emergency room admissions and deaths related to the consumption of these substances, particularly in cases where the use is inadvertent, which, together with the disadvantage of being substances that are difficult to detect in laboratory analysis, due to their rapid metabolization and variable concentration depending on the victim’s fluid intake, makes their identification and the determination of the range of involvement a challenge [123].

Recently, these benzodiazepines have been detected in counterfeit Xanax (alprazolam) tablets in Canada, which actually contained etizolam, flualprazolam, and flubromazolam. Consumption of these counterfeits is increasing in Central and South America, mainly in Chile and Paraguay; however, their use has spread to the United States, Australia, Singapore, and Malaysia, with counterfeits containing etizolam, flualprazolam, and phenazepam [124].

Currently, no studies have been identified that establish the inhalation incapacitation values (ICt_50_) or median lethal concentrations (LC_50_ or LCt_50_) for benzodiazepines in humans or animals. Although the safety data sheets classify these substances as “toxic if inhaled”, precise quantitative values are not specified [125,126]. On the other hand, the LD_50_ values of benzodiazepines in animals provide a clear picture of their acute toxicity (Table 3) [127,128,129,130]. This classification, together with their pharmacological profile, suggests that benzodiazepines would not be suitable for military purposes or use in contexts other than therapeutic ones [131].

Benzodiazepines generally have a high therapeutic index, which contributes to their relative safety when used as monotherapy. Despite this, their use carries with it a number of adverse reactions, including dizziness, drowsiness, headache, amnesia, decreased coordination, and their potential for addiction. Because of the latter consideration, conferring the risk of being consumed as a substance of abuse, 33 BZDs were included in Schedule IV of the 1971 Convention on Psychotropic Substances in 1984 [76].

In addition, there is one type of BZDs, triazolobenzodiazepines (such as triazolam), that have a narrower therapeutic margin due to their high potency, rapid onset, and short half-life. These characteristics increase the risk of adverse effects, such as paradoxical reactions, excessive sedation, respiratory depression, and cognitive impairment, especially in elderly patients or when used concomitantly with other central nervous system depressants [132,133].

#### 3.4.5. Synthetic Cannabinoids

Cannabinoid compounds are substances of various origins whose action is mediated by cannabinoid receptors. The endocannabinoid system is linked to various functions, including memory, behavior, nociception, gastrointestinal motility, motor control, and the immune system, to name a few [134]. The three major groups of cannabinoid receptor ligands are: those generated by mammals, endocannabinoids (anandamide and 2-arachidonylglycerol); those produced by the cannabis plant (*Cannabis sativa* L.), phytocannabinoids (D9-tetrahydrocannabinol, Δ9-THC); and those commercialized for recreational purposes, synthetic cannabinoids (SCs) [134].

**Figure 6:**
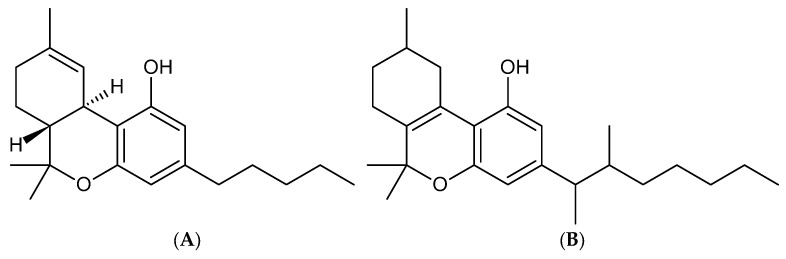
Chemical structure of tetrahydrocannabinol (THC) (**A**) and dimethylheptylpyran (DMHP) (**B**), which forms the chemical basis for EA-2233. EA-2233 is the O-acetate ester of DMHP and possesses three stereogenic centers, resulting in eight possible stereoisomers (EA-2233-1 to EA-2233-8), with varying R/S configurations.

The effects of the cannabis plant have been known for thousands of years; however, these effects at the neurochemical level have only recently been explored after the isolation and identification of phytocannabinoids. One of these identified molecules, D9-tetrahydrocannabinol (THC, shown in Figure 6), is the best known, with extensive information on its effects in humans [135]. Cannabis is anxiolytic, a sedative, and induces cognitive alterations, with the most common reasons for its use being the search for enjoyment, relaxation, euphoria, and increased sociability [134]. The recreational use of THC led to its classification as a Schedule I drug; however, numerous investigations on the use of phytocannabinoids (on THC and cannabidiol), foresee it as a possible treatment for nausea and pain, among other psychiatric conditions [135].

After the discovery of the structure of THC in 1960, a new type of cannabinoids, known as synthetic cannabinoids (SCs), were synthesized in 1970 for research on their functions and therapeutic utility as cannabinoid receptors [134,136]. However, since 2000, SCs have emerged within the illicit market as an alternative to phytocannabinoids, being sold as a “legal high” and abused as different variables of SCs [134,136]. SCs have been used recreationally probably since 2004, when they were initially marketed first as smokable herbs called “spice”, which were being sold on the internet [134]. However, 4 years later, herbal mixtures were discovered in regard to a German company, which contained JWH018, CP 47,497 and analogues of C6, C7, C8. Since then, this phenomenon has spread, and such substances are available from a wide variety of brands on websites [134].

SCs can affect a person’s mood, perception, thinking, memory, and attention, generating adverse effects, including anxiety, agitation, panic, dysphoria, and psychosis [136]. Toxicological reports describe different effects reported at the neurological (37%), psychiatric (25.6%), cardiorespiratory (44.1%), and gastrointestinal (13.2%) levels, with the most reported being tachycardia (40%), agitation (20%), somnolence, vomiting/nausea, and hallucinations [134].

Of the reported derivatives, more than 250 synthetic compounds have been found to interact with the endocannabinoid system these being synthesized in recent years by clandestine laboratories [135]. Currently, the number of reported SC-type NPSs in this family are 360 substances up until 2024, representing the totality of the synthetic agonists of the cannabinoid receptors reported by the UNODC [64].

Although cannabinoids, both natural and synthetic, are mainly used today for recreational purposes, there is a documented history of their evaluation in military contexts and in regard to their illicit use.

In search of the intoxicating properties of cannabis, in 1896, a group of researchers in Cambridge found a compound, which they called “narcotic red oil” [42]. Basically, “red oil” is a distilled cannabis resin that was the starting material for all the early studies on cannabis [43]. However, the attribution of its psychoactive effect turned out to be confusing. By 1940, the organic chemist, Roger Adams, had investigated the composition of that “red oil” and resolved this confusion by demonstrating that the oil was a complex mixture of compounds and not a single pure product, finding that pure cannabinol lacked intoxicating activity [137]. Adams then proposed that other compounds, not yet isolated at the time, would be truly responsible for the psychoactive properties of cannabis, laying the groundwork for the subsequent discovery and isolation of cannabidiol (CBD) and THC [43,137].

At the same time, in 1942, during World War II, the U.S. intelligence agency, OSS (Office of Strategic Services), which was in charge of coordinating espionage activities, sought to create a “truth serum” using some kind of substance that would override judgment and will in order to obtain enemy information [43,138]. The research committee led by Dr. Winfred Overholser and Harry Ansilinger, examined various substances, including alcohol, barbiturates, mescaline, and scopolamine, but due to the cognitive incoherencies, visions, and hallucinations generated by these substances that caused difficulties during interrogation, they were not considered [138]. In parallel, recent research by Adams on the psychoactive components of cannabis has opened up new possibilities for its use [43]. Taking advantage of Adams’ work on red oil, scientists at the OSS and the U.S. Chemical Corps succeeded in isolating their own concentrated cannabis extract, which they called “TD” (Truth Drug), a colorless and tasteless liquid obtained by the esterification of marijuana resin, promising to selectively suppress mental defenses without inducing perceptual alterations as severe as those of other previously tested drugs [138,139]. Despite this, the results indicated that its effects did not differ much from alcohol intoxication and the psychoactive effects of other substances, so they ended up suspending TD research and it was concluded that no substance demonstrates the ability to exert absolute control over the veracity of individuals, given the complexity of the psychoactive effects they induce [138,139,140].

Despite this, research on mental control or “incapacitation” continued. As mentioned, in 1953, although the MK-Ultra project was focused on the psychoactive properties of LSD-25, it also included several studies on cannabinoids [71]. Then, in the late 1950s and early 1960s, with the background gathered on cannabinoids, the U.S. Chemical Corps re-isolated its own “red oil” under the code EA-1476 [140,141]. Even though, EA-1476 produced a potent effect, researchers considered that the 40 µg/kg required to generate a 10–20% cognitive decline effect was considered outside the dose range for any practical military purpose [141]. Under this same premise on seeking more potent and incapacitating “non-lethal” analogs, in 1961, the effects of the synthetic cannabinoid 1,2-dimethylheptyl (DMHP, shown in Figure 6), assigned the code number EA-2233, which is the O acetate ester of dimethylheptylpyran, containing three stereogenic centers, leading to eight stereoisomers (EA-2233-1 to EA-2233-8) that differ according to the R/S configuration at these centers, discovered in 1949 by Adams during studies to elucidate the structure of THC, were investigated in depth [43,140,141].

At Edgewood Arsenal, Dr. James S. Ketchum and his team administered EA-2233 to military volunteers in oral doses ranging from 10 to 60 µg/kg. They observed that its psychoactive effects were significantly more intense and longer lasting than those of red oil, lasting up to 30 h and severely impairing motor coordination and cognitive function [141]. However, EA-2233 did not appear to be potent enough to be of military interest, as an oral dose of 60 µg/kg caused, at most, a cognitive decline of only 40% [141]. The ICT_50_ of DMHP is approximately 0.075 mg/kg in mice, while its intravenous LD_50_ is around 63 mg/kg in mice and about 10 mg/kg in dogs, values significantly higher than the incapacitating dose [140,142].

When the individual isomers of EA2233 became available in 1965, another series of tests was carried out with the improved version of this analogue, in which they were assigned the individual codes EA-2233-1 to EA-2233-8 [140,142]. After isolating the individual stereoisomers, EA-2233-2 was identified as the most potent isomer, as it was capable of inducing confusion, sedation, and hallucinogenic effects at doses ranging from 0.5 to 2.8 μg/kg (≈35–200 μg in a 70 kg adult), producing significant effects lasting up to 2–3 days. No documentation was found regarding its LD_50_ or ICT_50_ values [141].

These findings led Edgewood researchers to consider EA-2233-2 as “one of the most promising non-lethal incapacitating agents in their program” [141]. However, because these agents could occasionally induce severe hypotensive crises and hypothermia, they were ultimately not used as a weapon [141]. Additionally, for both political and pharmacological reasons, the initiative was abandoned, particularly after members of the press ridiculed the idea of the Army using an illegal recreational drug as a weapon of war [141].

The idea of developing a cannabis-based “incapacitating” weapon remains present today. In March 2017, the United States Patent and Trademark Office granted a patent to the California-based company, Saint Brand Cannabis, for the formulation of a cannabinoid agent (U.S. Patent No. 9,585,867 B2) intended to induce incapacitation and/or immobilization, for use in riot control and/or potential urban warfare scenarios. However, no further information has been made available regarding this development [140,143].

As previously mentioned, synthetic cannabinoids have evolved into a wide-ranging class of compounds. In recent decades, ultra-potent synthetic cannabinoids, such as AMB-FUBINACA and ADB-FUBINACA, have emerged [144,145]. Both compounds were originally synthesized in 2009 by the pharmaceutical company, Pfizer, for analgesic purposes. They share a modified indazole core, with side chains designed to optimize binding to cannabinoid receptors (CB_1_ and CB_2_), and exhibit a potency approximately 85 times greater than Δ^9^-THC [144]. Regarding their LD_50_ there is no precise data in the scientific literature for these SCs [134]. However, available sources, such as safety data sheets, suggest that AMB-FUBINACA has an estimated LD_50_ in rabbits via the dermal route of ~300.1 mg/kg, while ADB-FUBINACA has an estimate via the same route of ~1502 mg/kg [144,146].

In 2016, the New York City authorities witnessed a mass poisoning of 33 people with AMB-FUBINACA [145]. This episode was referred to as a “zombie outbreak” by local media due to the appearance of intoxicated users, who exhibited symptoms of severe central nervous system depression [145]. In 2017, Lam et al. [147] reported a case in Hong Kong involving a healthy 24-year-old man who used an electronic cigarette to inhale two drops of “VaporFi”, a product later found to contain AB-FUBINACA and ADB-FUBINACA. Approximately 30 min after inhalation, the patient developed drowsiness, confusion, and agitation, along with palpitations and vomiting. Upon arrival at the emergency department, he experienced a brief episode of supraventricular tachycardia, which resolved spontaneously [147].

Finally, in 2018, in Utah, United States, 52 people consumed what they believed to be medicinal CBD (cannabidiol) oil, but it was actually adulterated with a potent synthetic cannabinoid (4-cyano-Cumyl-butinaca), of which 31 people required medical attention [148]. Although it is unclear whether the last two cases were due to negligence by the clandestine manufacturer or intentional adulteration, they serve as an example of how these compounds can reach unsuspecting individuals without their knowledge [140,145,148].

On the other hand, in May 2024, it was reported by several media outlets that the UK Criminal Justice Workers union denounced an episode that occurred at HMP Swaleside prison in Kent, wherein 25 people, including guards and cooks, were affected after consuming Spice in their lunch [149,150]. It was later discovered that inmates working in the staff dining hall had allegedly added Spice to the food, causing nausea, dizziness, and a loss of coordination among personnel. A few months later, in September 2024, nine employees at the Stillwater Correctional Facility in Bayport, Minnesota, were hospitalized after coming into contact with an inmate handling unidentified substances. The substances were later confirmed to be SCs, and those affected reported symptoms including dizziness, nausea, and tachycardia [151,152].

These events, which have occurred mainly in penitentiary establishments, are due to the administration of MDMB-CHMICA and MDMB-4en-PINACA-type compounds impregnated in paper (letters, cards, or strips), using ethanol as a solvent [151,152].

According to the UNODC report entitled “Synthetic Cannabinoids in Herbal Products”, health problems associated with the use of synthetic cannabinoids include cardiovascular complications (tachycardia, arrhythmias), psychological disorders (anxiety, psychosis), and even the possible carcinogenic potential of some metabolites [153]. Regarding its international control, synthetic Δ^9^-THC and its stereoisomers were reclassified in Schedule II of the 1971 Convention on Psychotropic Substances following the 45th meeting of the Commission on Narcotic Drugs on 29 April 1991, recognizing its relatively low capacity for abuse and its therapeutic applications [154]. On the other hand, other synthetic cannabinoids are included in the list of substances in Schedule II of the United Nations Convention on Psychotropic Substances of 1971 [155].

A comparative summary of the incapacitating concentration (ICt_50_) and median lethal dose (LD_50_) values for different NPS families obtained from various animal models and administration routes is presented below. The details of these parameters are shown in Table 3.

**Table 3:** Summary of incapacity concentration and lethal doses of NPS substances.

NPS Family	Chemical Agent	Species	ICt_50_ Inhal. mg·min/m^3^	LD_50_ mg/kg	Sources
Lysergamides	LSD-25	Human	30 a 55	~0.2 (i.v.)	[66,67,69]
Phencyclidines	PCP	Rat	≈1000	16 (i.v.)	[82,83]
Fentanyl derivatives	Fentanyl	Monkey	NA	0.03 (i.v.)	[105]
	Carfentanil	Mouse	NA	18.75 (i.v.)	[110]
Benzodiazepines	Midazolam	Rat	NA	357 (i.v.)	[127]
	Alprazolam	Rat	NA	1200 (p.o.)	[128]
	Flunitrazepam	Rat	NA	415 (p.o.)	[129]
	Clonazolam	Rat	NA	~500 (p.o.)	[130]
Cannabinoids	DMHP	Mouse	≈0.075	~63 (i.v.)	[141,142]
	AMB-FUBINACA	Rabbit	NA	~300.1 (i.d.)	[156]
	ADB-FUBINACA	Rabbit	NA	~1502 (i.d.)	[157]

LSD-25: lysergic acid diethylamide, DMH: 1,2-dimethylheptyl, NA: not available.

## 4. Discussion

The chemical arsenal has evolved from rudimentary agents, such as the vesicants and choking agents used during World War I, to increasingly potent, lethal, and relatively easy-to-synthesize families, including the G, V, A, and Novichok series. This progression reveals two recurring patterns. The first pattern is the repurposing of substances originally designed for medical, industrial, or scientific purposes that were later adapted for warfare contexts [11]. As was the case with the initial use of industrial gases, such as chlorine and phosphagen, which led to research programs aimed at developing more specific compounds capable of interfering with critical physiological functions. The second pattern is the deliberate structural modification of pre-existing compounds with the aim of increasing their toxicity, persistence, or capacity for regulatory evasion [13,15]. The synthesis of tabun by IG Farben in 1936 served as the framework that initiated a new stage marked by the emergence of nerve agents, from which increasingly lethal derivatives were developed based on this core structure, such as sarin, VX, and, eventually, Novichok agents [13,15].

This same logic of chemical transformation and sophistication remains relevant today, especially with the emergence of new psychoactive substances (NPSs). Originally designed to circumvent international regulations on narcotic and psychotropic substances, these compounds have evolved into increasingly potent, versatile, and difficult-to-trace agents [7]. Their production, driven by clandestine laboratories with access to advanced synthesis and global distribution networks, including the digital black market, has enabled the emergence of, for example, synthetic opioids, such as nitazenes, which even surpass fentanyl in terms of potency and lethality [8].

Beyond the public health risk, as evidenced by the global rise in fatal intoxications, the danger of these substances lies in their potential for diversion to military or terrorist purposes. Many NPSs produce effects that go far beyond recreational use, including profound dissociation, persistent hallucinations, impaired judgment, and a loss of motor control [8,38]. These psychoactive effects are functionally indistinguishable from those that characterized so-called “incapacitating agents”, historically used for cognitive manipulation purposes [131]. However, as argued, the “incapacitating weapon” category is technically and ethically unsustainable, as all of the observed effects, including those of NPSs, compromise vital functions and may lead to death, thereby invalidating their classification as non-lethal weapons [40,48].

The psychotropic versatility of NPSs, capable of inducing dissociative states, paralysis, or respiratory collapse at minimal doses, and their continuous structural modification to evade legal controls, position them as concerning candidates for use as unconventional chemical weapons [38]. In Buenos Aires, Argentina, 20 people died and another 70 were hospitalized after consuming contaminated cocaine. After investigations were carried out and the respiratory symptoms were resolved using naloxone, it was discovered that this drug was mixed with carfentanil. Among the hypotheses proposed in response to this incident is the possibility that a drug trafficker aimed to discredit and undermine the business operation of a competing gang [158,159]. In addition, the cause of a cluster of severe intoxications caused by a new drug is often unknown to the medical community until it is first described in a case series [8,140]. Identifying the responsible agent requires collaboration among clinical laboratories, healthcare professionals, law enforcement, and chemists to disseminate timely information about causative substances. This underscores the difficulty of detecting these compounds and the ease with which they can be diverted from recreational use to potential chemical weapons applications [7,140].

As evidenced throughout this work, lysergamide, phencyclidine, fentanyl derivatives, benzodiazepines, and synthetic cannabinoids have been reported in documented incidents of hostile use, reinforcing their offensive potential in future harmful contexts. This point becomes more evident when considering the chemical evolution of these substances, which does not aim at temporary incapacitation, but rather at maximizing the physical and psychological impact through invisible and difficult-to-trace mechanisms [8,38].

The dangers posed by NPSs and their constant proliferation highlight the need for robust control frameworks. The Conventions on Narcotic Drugs and Psychotropic Substances establish the framework for the regulation of these substances for medical or research purposes, with the aim of preventing their illicit use [160]. However, these rigorously established measures do not compare to the regulations in place for substances that can be used as chemical weapons.

The Chemical Weapons Convention establishes a regulatory framework for the development, production, stockpiling, and transparency of such weapons, with a zero-tolerance approach to their use. Its definitions include not only toxic substances that affect humans or animals, but also precursors, munitions, equipment, or devices intended for use in connection with the employment of toxic chemicals for hostile purposes [161]. However, despite the CWC’s normative ambition, its implementation faces growing limitations in the face of the dynamism of contemporary chemistry. Schedule categorization, a central strategy of the treaty, was effective in the context of late 20th century chemical weapons, but has been proven to be too rigid in regard to the emergence of structurally novel substances [161]. Agents, such as Novichok, deliberately developed to avoid matching listed compounds, and emerging substances, like fentanyl derivatives or synthetic cannabinoids, exemplify how chemical innovation can surpass the capabilities of international regulatory frameworks [160,162,163].

In addition to these sections, the Chemical Weapons Convention introduces a key element in determining whether a substance can be considered a weapon or the intentionality of its use. Many substances have a legitimate intended use in everyday life, and it is generally presumed that they will be used for their declared purposes, such as in the case of organophosphate insecticides, in regard to the determination as to whether they should be included in the schedules.

However, the Convention also establishes that it is necessary to verify that these substances are used for their intended purpose, as in the case of organophosphate insecticides, to determine whether or not they should be listed [162].

These provisions outlined in the Convention raise a significant dilemma regarding NPSs: Can they be considered weapons if they were originally designed as recreational drugs or pharmaceuticals? Regulatory ambiguity creates a grey area when it comes to the potential use of NPSs for other purposes, particularly because the actual intent behind their use cannot always be proven or anticipated. The most prominent example of this alarming possibility is found in the case of fentanyl-derived opioids, which have historically served three general purposes: use as hospital analgesics, drugs of abuse, and chemical weapons [8].

Due to the benefits that several of these substances offer in research and clinical settings, it becomes challenging to consider them as potential threats, beyond their role as psychoactive drugs. For this reason, control measures should not only aim to include them in the schedules of the Chemical Weapons Convention but should also encourage the adoption of regulatory frameworks and legal alternatives that can effectively prohibit the circulation of potential NPSs via illicit markets.

One of the most used and researched approaches adopted by various countries is the implementation of a generic legislation model. This strategy seeks to regulate a common chemical structure and its possible modifications under the law, offering two key advantages, namely the ability to control a broader range of substances, and the avoidance of the need for constant legislative updates, as is the case when using lists [163].

As warned in the NATO report “Neuro-Cognitive Warfare: Inflicting Strategic Impact via Non-Kinetic Threat”, “*the brain will be the battlefield of the 21st century*” and “*human beings will be the contested domain*”. Furthermore, “*future conflicts will likely develop first in the digital realm and then physically, in the vicinity of centers of political and economic power*”. In addition to this, is the fact that artificial intelligence (AI) represents a great revolution, opening up new possibilities in multiple fields, including the transformation of scientific and technological disciplines, the economy, industry, and even medicine [164]. However, despite the revolutionary benefits of AI, this tool can have negative impacts on society if misused [165].

In 2020, the company, Collaborations Pharmaceuticals, which specializes in identifying new drug candidates for rare and infectious diseases, demonstrated how its AI model, MegaSyn^®^, could be adapted to design toxic compounds instead of medicines. In less than 6 h, more than 40,000 lethal substances had been generated [54,165,166]. Surprisingly, among the molecules generated was not only VX, the most toxic nerve agent known to date, but also newly designed compounds with equally plausible structures that, according to predicted median lethal dose (LD_50_) values, appeared to be even more toxic than existing chemical warfare agents [167]. Another article that demonstrates this possibility is “Catalyst: Curtailing the scalable supply of fentanyl through the use of chemical AI”, which shows how AI can identify new ways of synthesizing fentanyl, using conventional methods, from known, regulated precursor substances, and non-conventional methods, from unregulated and even easy-to-obtain precursor substances [168].

Based on this premise, AI could help circumvent legislation, which is not equipped to react quickly to new technological discoveries. These challenges posed by AI are already being considered by relevant institutions; in fact, the danger of its use in the creation and synthesis of opioid-derived NPSs, such as fentanyl or nitazenes, which could be much more potent and capable of circumventing legal controls, is anticipated [8]. This highlights the need to explore new strategies for addressing these emerging technological threats, with the aim of preventing the harmful use of AI in the creation of chemical substances. Fortunately, AI still has limitations as it is based only on available knowledge. Therefore, it is possible that the creation of new AI-designed drugs or synthesis pathways will not be possible in practice.

## 5. Conclusions

This work shows that the line between traditional chemical agents and new psychoactive substances (NPSs) can be blurred, ranging from the well-known nerve agents in the G, V, and A series to emerging compounds, such as fentanyl derivatives, or more potent cannabinoid compounds. Although these groups differ in regard to their origin and initial purposes, they share four critical features that enhance their potential for hostile use: (i) high toxic potency with low effective doses, (ii) relative ease of synthesis or structural modification through accessible chemical routes, (iii) rapid circulation via illicit markets and online channels, and (iv) regulatory gaps that allow for their production and distribution almost in real time with respect to control efforts.

Historical analysis, from the introduction of blister agents in World War I to recent events linked to Novichok, shows that chemical innovations often precede regulatory frameworks. NPSs amplify this trend: their speed of emergence exceeds the capacity of current control lists, while their therapeutic ambivalence complicates classification decisions. If this dynamic continues, states and non-state actors will have an increasingly wide range of substances with potential for military or terrorist use, increasing the likelihood of mass poisoning incidents and hindering forensic attribution.

Given this scenario, it is essential to strengthen a three-pronged approach. First, the Chemical Weapons Convention and national legislation must be updated in a flexible and forward-looking manner, incorporating criteria based on mechanisms of action and risk profiles rather than static lists of compounds. Second, the consolidation of toxicovigilance systems and forensic laboratories should occur with the capacity to rapidly identify recently synthesized analogues, supported by international analytical and spectral data exchange networks. Third, the promotion of codes of conduct and ethics committees in chemical and pharmaceutical research should be established that anticipate diversions of dual-use substances, together with interdisciplinary training programs that integrate public health, security, and foreign policy.

In conclusion, the convergent evolution of conventional chemical agents and NPSs requires a shift from the adoption of a reactive to a preventive approach, based on dynamic regulatory science, multilateral cooperation, and continuous technological surveillance. Only coordinated action, ranging from the laboratory bench to diplomatic forums, can mitigate the risk of NPSs becoming the next generation of chemical weapons.

## Figures and Tables

**Table 1:** Estimates of relative potency in humans of some agents.

Generation	Chemical Agent	LCt_50_ (Inhal.) mg·min/m^3^	LD_50_ p.c. mg/kg (Liquid)	LCt_50_ p.c. (Vapor) mg·min/m^3^	Sources
First	Phosphogen	2000	NA	NA	[23]
	Lewisite	1200	35–40	99,000	[24,25]
	Mustard Gas (HD)	1500	100	10,000	[26,27]
Second	Tabun (GA)	70	~21.4	15,000	[28]
	Sarin (GB)	35	~24	10,000	[29]
	Soman (GD)	35	~5	2500	[30]
Third	VX	30	0.07	150	[31]
Fourth	A-230	1.9–3	0.6	NA	[22]
	A-232 (Novichok 5)	7	5	NA	[32]

HD: bis(2-chloroethyl) sulfide; GA: ethyl-(dimethylphosphoramido)cyanidate; GB: isopropyl methylphosphonofluoridate; GD: (3,3-dimethylbutan-2-yl) methylphosphonofluoridate; GF: cyclohexyl methylphosphonofluoridate; VX: S-[(2-diisopropylamino)ethyl]-O-ethyl methylphosphonothioate; VE: S-[(2-diethylamino)ethyl]-O-ethyl methylphosphonothioate; VM: S-[(2-diethylamino)ethyl]-O-methyl methylphosphonothioate; VG: S-[(2-diisopropylamino)ethyl]-O-isobutyl methylphosphonothioate; A-230: N-2-diethylaminomethylacetamido methylphosphonofluoridate; Novichok 5: methyl-(1-(diethylamino)ethylidene)phosphoramidofluoridate. NA: not available.

**Table 2:** Chemical agents used as chemical weapons. Adapted from the Organization for the Prohibition of Chemical Weapons [20].

Type of Chemical Agent	Examples of Agents	Method of Dispersion	Mode of Action	Effects
Asphyxiants	Chlorine (Cl_2_), Phosgene (CG), Diphosgene (DP), Chloropicrin (PS).	Gas	Absorption through the lungs.	Accumulation of fluid in the lungs that causes irritation to the nose, throat and airways, making breathing difficult, which can lead to asphyxia and death.
Vesicants	Sulfur Mustard (HHD), Nitrogen Mustard (HN), Lewisite (L), Phosgene Oxime (CX).	Liquid, Aerosol, Vapor, and Dust	Absorption through the lungs and skin.	Irritation of the skin and mucous membranes, causing blistering of the exposed surface, and respiratory tract, with the risk of being life threatening.
Hemotoxicants	Hydrogen Cyanide (AC), Cyanogen Chloride (CK), Arsine (SA).	Gas	Absorption through the lungs and skin; inhibition of the enzyme, cytochrome C oxidase.	They interfere with the ability to transport oxygen in the blood, causing hypoxia.
Neurotoxicants	Sarin (GB), Soman (GD), Tabun (GA), VX, Novichok (A-230).	Liquid, Aerosol, Vapor, and Dust	Absorption through the lungs (G-series); skin contact (V-series); inhibition of acetyl cholinesterase.	They generate hyperstimulation of the central nervous system, causing convulsions, muscle paralysis, respiratory distress, dizziness, confusion, and, in extreme cases, death.
Riot Control Agents (RCAs)	Pepper Gas (OC), Tear Gas (CS).	Liquid, Aerosol, Gas	Absorption through lungs, skin, and eyes.	Irritation of mucous membranes and activation of pain receptors in eyes, nose, and throat. Causes excessive tearing, shortness of breath, coughing, excessive salivation, and nasal discharge.

## Data Availability

Not applicable.

## Appendix A

The Chemical Weapons Convention (CWC) establishes three schedules in the Annex on Chemicals, which outline the categories of substances that must be controlled, monitored, or destroyed. These schedules enable the graduated control of chemical substances, and their precursors based on their potential to be used in the production of chemical weapons or in other activities prohibited by the Convention [19].

**Table A1:** Lists of chemicals according to the Annex on Chemicals in the Chemical Weapons Convention (CWC). Adapted from [19].

Schedule	Description	Guidelines
Schedule 1	Highly hazardous chemicals developed or used as chemical weapons.	Developed, produced, stockpiled, or used as chemical weapons. Similar in structure to substances already included. Lethal or incapacitating toxicity.
Schedule 2	Chemical substances with the potential to be used in the production of chemical weapons.	May be used as precursors or in the production of Schedule 1 substances. They are highly toxic or incapacitating.
Schedule 3	Chemicals and precursors that can be used for commercial purposes, but require control.	Used in industry, but with potential for diversion to prohibited activities. Not directly associated with chemical weapons production.

**Table A2:** Schedule 1 of toxic chemicals and precursors as listed in the Annex to the Chemical Weapons Convention (CWC). Adapted from [19].

Toxic Chemicals		
Number	Substance Name	Number CAS
(1)	O-Alkyl (≤C10, incl. cycloalkyl) alkyl (Me, Et, n-Pr or i-Pr)-phosphonofluoridates	
Example	Sarin: O-Isopropyl methylphosphonofluoridate	(107-44-8)
	Soman: O-Pinacolyl methylphosphonofluoridate	(96-64-0)
(2)	O-Alkyl (≤C10, incl. cycloalkyl) N,N-dialkyl (Me, Et, n-Pr or i-Pr) phosphoramidocyanidates	
Example	Tabun: O-Ethyl N,N-dimethyl phosphoramidocyanidate	(77-81-6)
(3)	O-Alkyl (H or ≤C10, incl. cycloalkyl) S-2-dialkyl (Me, Et, n-Pr or i-Pr)-aminoethyl alkyl (Me, Et, n-Pr or i-Pr) phosphonothiolates and corresponding alkylated or protonated salts	
Example	VX: O-Ethyl S-2-diisopropylaminoethyl methyl phosphonothiolate	(50782-69-9)
(4)	Sulfur mustards:	
	2-Chloroethylchloromethylsulfide	(2625-76-5)
	Mustard gas: Bis(2-chloroethyl)sulfide	(505-60-2)
	Bis(2-chloroethylthio)methane	(63869-13-6)
	Sesquimustard: 1,2-Bis(2-chloroethylthio)ethane	(3563-36-8)
	1,3-Bis(2-chloroethylthio)-n-propane	(63905-10-2)
	1,4-Bis(2-chloroethylthio)-n-butane	(142868-93-7)
	1,5-Bis(2-chloroethylthio)-n-pentane	(142868-94-8)
	Bis(2-chloroethylthiomethyl)ether	(63918-90-1)
	O-Mustard: Bis(2-chloroethylthioethyl)ether	(63918-89-8)
(5)	Lewisites:	
	Lewisite 1: 2-Chlorovinyldichloroarsine	(541-25-3)
	Lewisite 2: Bis(2-chlorovinyl)chloroarsine	(40334-69-8)
	Lewisite 3: Tris(2-chlorovinyl)arsine	(40334-70-1)
(6)	Nitrogen mustards:	
	HN1: Bis(2-chloroethyl)ethylamine	(538-07-8)
	HN2: Bis(2-chloroethyl)methylamine	(51-75-2)
	HN3: Tris(2-chloroethyl)amine	(555-77-1)
(7)	Saxitoxin	(35523-89-8)
(8)	Ricin	(9009-86-3)
(13)	P-alkyl (H or ≤C10, incl. cycloalkyl) N-(1-(dialkyl(≤C10, incl. cycloalkyl)amino))alkylidene(H or ≤C10, incl. cycloalkyl) phosphonamidic fluorides and corresponding alkylated or protonated salts	
Example	N-(1-(di-n-decylamino)-n-decylidene)-P-decylphosphonamidic fluoride	(2387495-99-8)
	Methyl-(1-(diethylamino)ethylidene)phosphonamidofluoridate	(2387496-12-8)
(14)	O-alkyl (H or ≤C10, incl. cycloalkyl) N-(1-(dialkyl(≤C10, incl. cycloalkyl)amino))alkylidene(H or ≤C10, incl. cycloalkyl) phosphoramidofluoridates and corresponding alkylated or protonated salts	
Example	O-n-Decyl N-(1-(di-n-decylamino)-n-decylidene)phosphoramidofluoridate	(2387496-00-4)
	Methyl (1-(diethylamino)ethylidene)phosphoramidofluoridate	(2387496-04-8)
	Ethyl (1-(diethylamino)ethylidene)phosphoramidofluoridate	(2387496-06-0)
(15)	Methyl-(bis(diethylamino)methylene)phosphonamidofluoridate	(2387496-14-0)
(16)	Carbamates (quaternaries and bisquaternaries of dimethylcarbamoyloxypyridines)	
	Quaternaries of dimethylcarbamoyloxypyridines:	
	1-[N,N-dialkyl(≤C10)-N-(n-(hydroxyl, cyano, acetoxy)alkyl(≤C10)) ammonio]-n-[N-(3-dimethylcarbamoxy-α-picolinyl)-N,N-dialkyl(≤C10) ammonio]decane dibromide (n = 1–8)	
Example	1-[N,N-dimethyl-N-(2-hydroxy)ethylammonio]-10-[N-(3-dimethylcarbamoxy-α-picolinyl)-N,N-dimethylammonio]decane dibromide	(77104-62-2)
	Bisquaternaries of dimethylcarbamoyloxypyridines:	
	1,n-Bis[N-(3-dimethylcarbamoxy-α-picolyl)-N,N-dialkyl(≤C10) ammonio]-alkane-(2,(n-1)-dione) dibromide (n = 2–12)	
Example	Dibromuro de 1,10-bis[N-(3-dimetilcarbamoxi-α-picolil)-N-etil-N-metilamonio]decano-2,9-diona)	(77104-00-8)
**Precursors**		
**Number**	**Precursor name**	**Number CAS**
(9)	Alkyl (Me, Et, n-Pr or i-Pr) phosphonyldifluorides	
Example	DF: Methylphosphonyldifluoride	(676-99-3)
(10)	O-Alkyl (H or ≤C10, incl. cycloalkyl) O-2-dialkyl (Me, Et, n-Pr or i-Pr)-aminoethyl alkyl (Me, Et, n-Pr or i-Pr) phosphonites and corresponding alkylated or protonated salts	
Example	QL: O-Ethyl O-2-diisopropylaminoethyl methylphosphonite	(57856-11-8)
(11)	Chlorosarin: O-Isopropyl methylphosphonochloridate	(1445-76-7)
(12)	Chlorosoman: O-Pinacolyl methylphosphonochloridate	(7040-57-5)

**Table A3:** Schedule 2 of toxic chemicals and precursors as listed in the Annex to the Chemical Weapons Convention (CWC).

Toxic Chemicals		
Number	Substance Name	Number CAS
(1)	Amiton: O,O-Diethyl S-[2-(diethylamino)ethyl] phosphorothiolate	(78-53-5)
	and corresponding alkylated or protonated salts	
(2)	PFIB: 1,1,3,3,3-Pentafluoro-2-(trifluoromethyl)-1-propene	(382-21-8)
(3)	BZ: 3-Quinuclidinyl benzilate	(6581-06-2)
**Precursors**		
**Number**	**Precursor name**	**Number CAS**
(4)	Chemicals, except for those listed in Schedule 1, containing a phosphorus atom to which is bonded one methyl, ethyl, or propyl (normal or iso) group, but not further carbon atoms	
Example	Methylphosphonyl dichloride	(676-97-1)
	Dimethyl methylphosphonate	(756-79-6)
Exemption: Fonofos	O-Ethyl S-phenyl ethylphosphonothiolothionate	(944-22-9)
(5)	N,N-Dialkyl (Me, Et, n-Pr or i-Pr) phosphoramidic dihalides	—
(6)	Dialkyl (Me, Et, n-Pr or i-Pr) N,N-dialkyl (Me, Et, n-Pr or i-Pr)-phosphoramidates	—
(7)	Arsenic trichloride	(7784-34-1)
(8)	2,2-Diphenyl-2-hydroxyacetic acid	(76-93-7)
(9)	Quinuclidin-3-ol	(1619-34-7)
(10)	N,N-Dialkyl (Me, Et, n-Pr or i-Pr) aminoethyl-2-chlorides and corresponding protonated salts	—
(11)	N,N-Dialkyl (Me, Et, n-Pr or i-Pr) aminoethane-2-ols and corresponding protonated salts	
Exemptions:	N,N-Dimethylaminoethanol	(108-01-0)
	and corresponding protonated salts	
	N,N-dietilaminoetanol	(100-37-8)
	and corresponding protonated salts	
(12)	N,N-Dialkyl (Me, Et, n-Pr or i-Pr) aminoethane-2-thiols and corresponding protonated salts	—
(13)	Thiodiglycol: Bis(2-hydroxyethyl)sulfide	(111-48-8)
(14)	Pinacolyl alcohol: 3,3-Dimethylbutan-2-ol	(464-07-3)

**Table A4:** Schedule 3 of toxic chemicals and precursors as listed in the Annex to the Chemical Weapons Convention (CWC).

Toxic Chemicals		
Number	Substance Name	Number CAS
(1)	Phosgene: Carbonyl dichloride	(75-44-5)
(2)	Cyanogen chloride	(506-77-4)
(3)	Hydrogen cyanide	(74-90-8)
(4)	Chloropicrin: Trichloronitromethane	(76-06-2)
**Precursors**		
**Number**	**Precursor name**	**Number CAS**
(5)	Phosphorus oxychloride	(10025-87-3)
(6)	Phosphorus trichloride	(7719-12-2)
(7)	Phosphorus pentachloride	(10026-13-8)
(8)	Trimethyl phosphite	(121-45-9)
(9)	Triethyl phosphite	(122-52-1)
(10)	Dimethyl phosphite	(868-85-9)
(11)	Diethyl phosphite	(762-04-9)
(12)	Sulfur monochloride	(10025-67-9)
(13)	Sulfur dichloride	(10545-99-0)
(14)	Thionyl chloride	(7719-09-7)
(15)	Ethyldiethanolamine	(139-87-7)
(16)	Methyldiethanolamine	(105-59-9)
(17)	Triethanolamine	(102-71-6)
